# Genome Sequences of SARS-CoV-2 Strains from the Republic of Moldova

**DOI:** 10.1128/mra.01132-22

**Published:** 2022-12-12

**Authors:** Mariana Ulinici, Martín Soñora, Emanuele Orsini, Danilo Licastro, Simeone Dal Monego, Mihail Todiras, Ludmila Lungu, Stanislav Groppa, Alessandro Marcello

**Affiliations:** a Nicolae Testemitanu State University of Medicine and Pharmacy, Chisinau, Republic of Moldova; b Alfa Diagnostica Laboratory, Chișinău, Republic of Moldova; c Biomolecular Simulation Group, Institut Pasteur de Montevideo, Montevideo, Uruguay; d Laboratory of Molecular Virology, International Centre for Genetic Engineering and Biotechnology, Trieste, Italy; e AREA Science Park, Trieste, Italy; DOE Joint Genome Institute

## Abstract

The whole-genome sequences of 15 severe acute respiratory syndrome coronavirus 2 (SARS-CoV-2) strains from nasopharyngeal swab samples collected in the Republic of Moldova in June 2020 to September 2021 were determined. Little variability was observed in the early stages, when mostly clade 19A was circulating, followed by clade 20B. Later, multiple introductions of SARS-CoV-2 lineages B.1.1., B.1.1.7, and B.1.1.525 were detected. The B.1.1.7 lineage became predominant between December 2020 and June 2021, followed by the Delta variant.

## ANNOUNCEMENT

The Republic of Moldova is a small Eastern European country with a population of 3.6 million (1). On 7 March 2020, a 48-year-old woman who returned from Italy represented the first recorded case of severe acute respiratory syndrome coronavirus 2 (SARS-CoV-2) (family *Coronaviridae*, genus *Betacoronavirus*), as confirmed by real-time reverse transcription-polymerase chain reaction (RT-PCR) (2). In 1 month, the number of cases increased to 965, with 854 cases being estimated to be due to local transmission and 111 being imported cases (3). To date, there have been 590,811 confirmed coronavirus disease 2019 (COVID-19) cases and 11,858 deaths (4).

This study involves the full genome sequencing of 15 SARS-CoV-2 isolates that were collected between June 2020 (4 samples) and September 2021 (11 samples) at the State University of Medicine and Pharmacy (SUMPh) in the Republic of Moldova, in collaboration with Alfa Diagnostica Laboratory. Nasopharyngeal swab samples were taken from suspected cases that met the guidelines of the Moldovan government (5).

We made an effort to make the sequences publicly available as soon as possible, with an average collection-to-submission time of 68 days. This report aims at framing these data in the context of SARS-CoV-2 molecular evolution in the country and in Europe in general.

Samples were collected in 1,000 μL transport medium (product number C-8885; Vector-Best). The nucleic acid extraction from clinical samples was carried out using a DNA/RNA manual extraction kit (product number C-8896; Vector-Best). For diagnosis, a SARS-CoV-2/SARS-CoV multiplex real-time PCR detection kit (product number R3-P436-23/9EU; DNA-Technology Research & Production) was used with primers targeting the E gene and the N gene. A CFX96 Touch real-time thermal cycler system (Bio-Rad) was used for amplification. Samples with cycle threshold (*C_T_*) values of <30 were considered for full genome sequencing. RNA from selected samples was aliquoted, anonymized, and sent at −80°C to the International Centre for Genetic Engineering and Biotechnology (ICGEB).

Library preparation and sequencing were performed as described previously (6–9). Briefly, a Qubit 2.0 fluorimeter (Thermo Fisher Scientific, USA) and 2100 Bioanalyzer (Agilent Technologies, USA) were used to assess RNA quantities and qualities, respectively. One hundred nanograms of RNA was processed with the Swift Amplicon SARS-CoV-2 research panel (Swift Biosciences, USA). Sequencing was conducted using an Illumina MiSeq sequencer with the standard protocol for paired-end 150-bp reads.

Quality control of raw data was conducted using FastQC v.0.11.9 (https://www.bioinformatics.babraham.ac.uk/projects/fastqc). For adapter removal and read trimming, the Primerclip trimming tool and adapter sequences for Swift Biosciences Accel-Amplicon panels were used. Genome assembly was conducted using dedicated Swift dockerized data analysis guidelines (10, 11). All tools were run with default parameters unless otherwise specified. For each sample, the sequencing parameters are listed in Table 1.

**TABLE 1:** Genomic parameters of SARS-CoV-2 strains from the Republic of Moldova

Sample no.	Strain^*a*^	GISAID accession no.	GenBank accession no.	SRA accession no.	Patient location	Sample collection date (day/mo/yr)	GISAID clade	Nextstrain clade	No. of raw reads	Genome size (bp)	Coverage depth (×)	GC content (%)	Pango lineage^*b*^	Amino acid substitutions
1	hCoV-19/Moldova/ICGEB_MD6/2020	EPI_ISL_516938	OP783441	SRX14472462	Rabnita, Moldova	18/6/2020	GR	20B	1,111,830	29,900	5,570	45.20	B.1.1 (Pango v.3.1.20)	Spike D614G, N G204R, N R203K, NS3 S165F, NSP2 L410F, NSP3 N1785D
2	hCoV-19/Moldova/ICGEB_MD7/2020	EPI_ISL_516936	OP783440	SRX14472461	Straseni, Moldova	18/6/2020	GR	20B	1,050,968	29,886	8,149	45.00	B.1.1 (Pango v.3.1.20)	
3	hCoV-19/Moldova/ICGEB_MD4/2020	EPI_ISL_516935	OP783439	SRX14472460	Balti, Moldova	17/6/2020	G	20A	1,080,708	21,179	5,312	45.50	B.1	
4	hCoV-19/Moldova/ICGEB_MD1/2020	EPI_ISL_516934	OP783438	SRX14472459	Chisinau, Moldova	17/6/2020	GR	20B	932,600	29,867	7,583	45.50	B.1.1 (Pango v.3.1.20)	
5	hCoV-19/Moldova/ICGEB_818767_S16/202	EPI_ISL_3886568	OP783436	SRX13323315	Cimislia, Moldova	11/5/2021	GR	20I/501Y.V1	929,928	29,540	8,149	45.30	B.1.1.7 (Pango v.3.1.20), Alpha (B.1.1.7-like) (Scorpio)	Spike A570D, spike D614G, spike D1118H, spike P681H, spike S982A, spike T716I, N D3L, N G204R, N R203K, N S235F, NS3 D27Y, NS8 Q27stop, NS8 R52I, NS8 Y73C, NSP3 A890D, NSP3 I1412T, NSP3 T183I, NSP6 G188C, NSP12 P323L, NSP12 V405A
6	hCoV-19/Moldova/ICGEB_819913_S18/2021	EPI_ISL_3903719	OP783437	SRX13323314	Chisinau, Moldova	12/5/2021	GR	20I/501Y.V1	513,580	29,542	8,149	45.80	B.1.1.7 (Pango v.3.1.20), Alpha (B.1.1.7-like) (Scorpio)	Spike A570D, spike D614G, spike D1118H, spike N501Y, spike P681H, spike S982A, spike T716I, spike V445I, N D3L, N G204R, N R203K, N S235F, NS7a P84A, NS8 Q27stop, NS8 R52I, NS8 Y73C, NSP2 R362H, NSP3 A890D, NSP3 D410G, NSP3 I1412T, NSP3 T183I, NSP12 P323L
7	hCoV-19/Moldova/ICGEB_809515_S10/2021	EPI_ISL_3886566	OP783435	SRX13323313	Chisinau, Moldova	5/5/2021	GR	20I/501Y.V1	435,738	29,540	8,149	45.90	B.1.1.7 (Pango v.3.1.20), Alpha (B.1.1.7-like) (Scorpio)	Spike A570D, spike D614G, spike D1118H, spike P681H, spike S982A, spike T716I, N D3L, N G204R, N R203K, N S235F, NS8 Q27stop, NS8 R52I, NS8 Y73C, NSP3 I1412T, NSP3 T183I, NSP5 Y101C, NSP12 P323L, NSP13 T115I
8	hCoV-19/Moldova/ICGEB_812387_S15/2021	EPI_ISL_3886564	OP783434	SRX13323312	Dubasari, Moldova	7/5/2021	GR	20I/501Y.V1	551,564	29,541	8,149	46.10	B.1.1.7 (Pango v.3.1.20), Alpha (B.1.1.7-like) (Scorpio)	Spike A570D, spike D614G, spike D1118H, spike N501Y, spike P681H, spike S982A, spike T716I, N D3L, N G204R, N R203K, N S235F, NS8 Q27stop, NS8 R52I, NS8 Y73C, NSP3 A890D, NSP3 I1412T, NSP3 T183I, NSP12 P323L
9	hCoV-19/Moldova/ICGEB_819835_S17/2021	EPI_ISL_3886562	OP783433	SRX13323311	Causeni, Moldova	12/5/2021	GR		688,252	29,541	8,149	46.40	B.1.1.523 (Pango v.3.1.20)	Spike D614G, spike D839V, spike E780A, spike F306L, spike N211K, spike T1027I, M I82T, N D22Y, N G204R, N G212C, N R203K, NSP2 N269D, NSP3 F1496L, NSP3 M84V, NSP3 R1297I, NSP4 T114I, NSP5 V303I, NSP6 V84F, NSP10 T111I, NSP12 P323L, NSP12 S229N, NSP13 L455M, NSP13 P77L, NSP15 V338A
10	hCoV-19/Moldova/ICGEB_797186_S3/2021	EPI_ISL_3886560	OP783432	SRX13323323	Chisinau, Moldova	24/4/2021	GR	20I/501Y.V1	608,708	29,541	8,149	45	B.1.1.7 (Pango v.3.1.20), Alpha (B.1.1.7-like) (Scorpio)	Spike A570D, spike D614G, spike D1118H, spike P681H, spike S982A, spike T716I, N D3L, N G204R, N R203K, N S235F, NS3 I232V, NS8 Q27stop, NS8 R52I, NS8 Y73C, NSP3 A338T, NSP3 A890D, NSP3 I1412T, NSP3 T183I, NSP12 P323L
11	hCoV-19/Moldova/ICGEB_823299_S20/2021	EPI_ISL_3886557	OP783431	SRX13323320	Stefan Voda, Moldova	14/5/2021	GR	20I/501Y.V1	817,382	29,541	8,149	45.50	B.1.1.7 (Pango v.3.1.20), Alpha (B.1.1.7-like) (Scorpio)	Spike A570D, spike D614G, spike D1118H, spike G1219C, spike P681H, spike S982A, spike T716I, N D3L, N G204R, N R203K, N S235F, NS8 Q27stop, NS8 R52I, NS8 Y73C, NSP3 A890D, NSP3 I1412T, NSP3 T183I, NSP6 A51V, NSP12 P323L
12	hCoV-19/Moldova/ICGEB_810217_S12/2021	EPI_ISL_3886556	OP783430	SRX13323319	Chisinau, Moldova	5/5/2021	GR	20I/501Y.V1	518,006	29,534	8,149	46	B.1.1.7 (Pango v.3.1.20), Alpha (B.1.1.7-like) (Scorpio)	Spike A570D, spike D614G, spike D1118H, spike H69del, spike P681H, spike S982A, spike T716I, spike V70del, N D3L, N G204R, N R203K, N S235F, NS8 Q27stop, NS8 R52I, NS8 Y73C, NSP3 A890D, NSP3 I1412T, NSP3 T183I, NSP9 T19I, NSP12 P323L
13	hCoV-19/Moldova/ICGEB_797065_S2/2021	EPI_ISL_3886554	OP783429	SRX13323318	Chisinau, Moldova	24/4/2021	GR	20I/501Y.V1	553,028	29,540	8,149	45.60	B.1.1.7 (Pango v.3.1.20), Alpha (B.1.1.7-like) (Scorpio)	Spike A570D, spike D614G, spike D1118H, spike P681H, spike S982A, spike T716I, N D3L, N G204R, N R203K, N S235F, NS3 T271I, NS7b F9S, NS8 Q27stop, NS8 R52I, NS8 Y73C, NSP3 I1412T, NSP3 T183I, NSP12 P323L
14	hCoV-19/Moldova/ICGEB_798119_S4/2021	EPI_ISL_3886552	OP783428	SRX13323310	Chisinau, Moldova	26/4/2021	GR	20I/501Y.V1	995,082	29,689	8,149	44.80	B.1.1.7 (Pango v.3.1.20), Alpha (B.1.1.7-like) (Scorpio)	Spike A570D, spike D614G, spike D1118H, spike P631S, spike P681H, spike S982A, spike T716I, spike W152R, N D3L, N G204R, N R203K, N S235F, NS8 Q27stop, NS8 R52I, NS8 Y73C, NSP3 A890D, NSP3 I1412T, NSP3 S1717L, NSP3 T183I, NSP12 P323L, NSP14 K165R, NSP16 P236L
15	hCoV-19/Moldova/ICGEB_796301_S1/2021	EPI_ISL_3886549	OP783427	SRX13323309	Hincesti, Moldova	24/4/2021	GR	20I/501Y.V1	621,908	29,541	8,149	45.80	B.1.1.7 (Pango v.3.1.20), Alpha (B.1.1.7-like) (Scorpio)	Spike A570D, spike D614G, spike D1118H, spike P681H, spike S982A, spike T716I, N D3L, N G204R, N R203K, N S235F, NS7a P34S, NS8 Q27stop, NS8 R52I, NS8 Y73C, NSP2 L550F, NSP2 S203G, NSP3 A890D, NSP3 I1412T, NSP3 T183I, NSP3 V473F, NSP6 L37F, NSP8 T187I, NSP12 P323L, NSP16 D179G

a For the amino acid substitutions, the original/reference strain (hCoV-19/Wuhan/WH01/2019 (EPI_ISL_402125)) was used for comparison.

b Pango v.3.1.20 is dated 28 February 2022.

**FIG 1:**
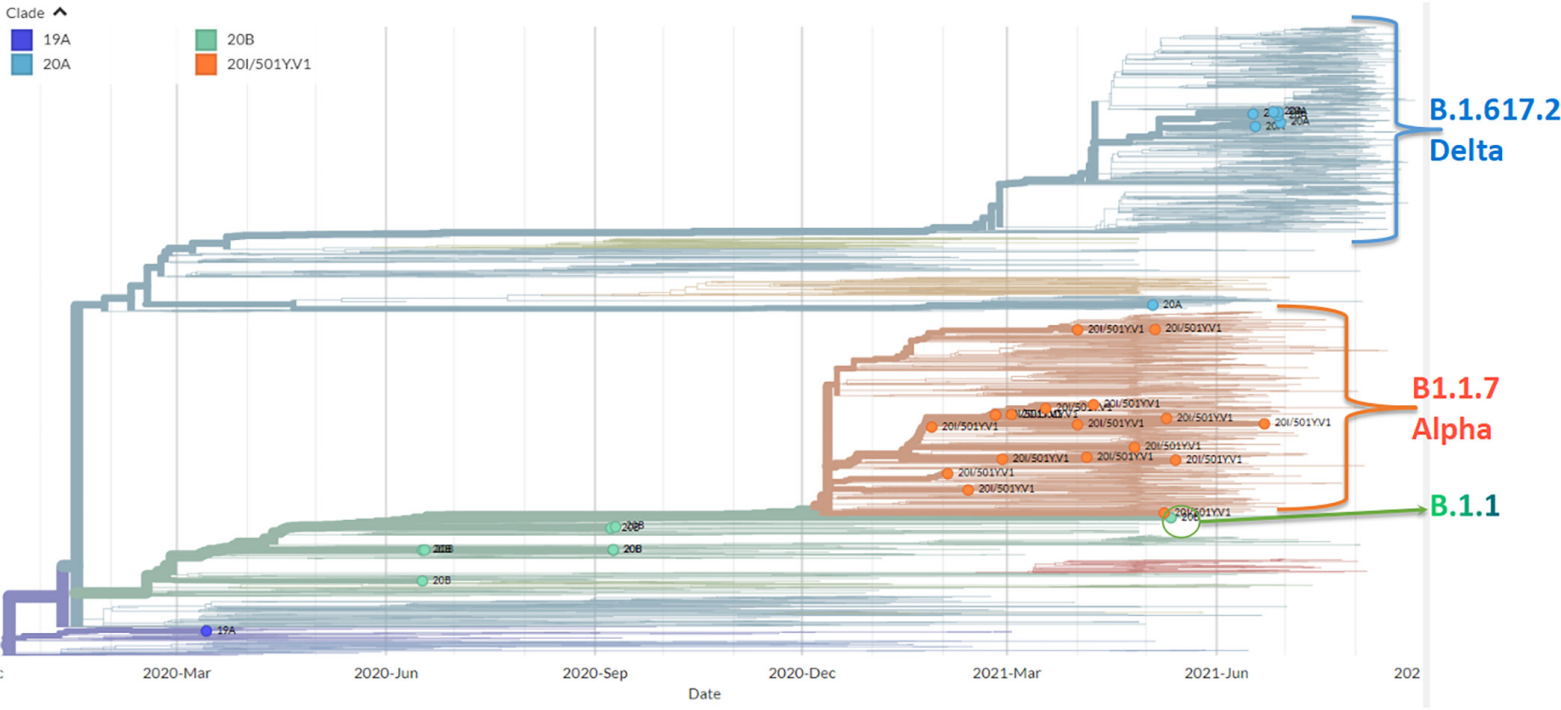
Phylogenetic tree, in rectangular view, for European SARS-CoV-2 strains, including the 15 strains collected in the Republic of Moldova.

Phylogenetic analyses were performed through the Nextstrain bioinformatics platform (12). A Moldova-focused country-level subsampling strategy was used in the context of the Nextregions/Europe data set (updated to 1 September 2021); the reference strain hCoV-19/Wuhan/WH01/2019 (GISAID accession number EPI_ISL_402125) was used as the original root. From this analysis, 4 clades were identified, i.e., 19A, 20A, 20B, and 20I/501Y.V1 (Table 1 and Fig. 1). Although limited in numbers, the present study enriches the collection of sequences from Moldova and provides a framework for enhanced molecular epidemiology in the country.

The study was reviewed and approved by the Research Ethics Committee of Nicolae Testemitanu SUMPh (protocol number 3/24.01.22). All samples were coded, and the information obtained from the study participants was kept confidential. Sample selection followed WHO recommendations for countries with limited resources (13).

### Data availability.

The metadata and the coding-complete genome sequences of all 15 samples were submitted to the GISAID (www.gisaid.org) database and can be found under GISAID accession number EPI_SET_221017uv (https://doi.org/10.55876/gis8.221017uv). Sequences are also available in the NCBI database under BioSample accession numbers SAMN26687402, SAMN26687401, SAMN26687400, SAMN26687399, SAMN23672914, SAMN23672913, SAMN23672912, SAMN23672911, SAMN23672910, SAMN23672909, SAMN23672906, SAMN23672905, SAMN23672904, SAMN23672901, and SAMN23672900. The raw reads were deposited in the NCBI Sequence Read Archive (SRA) database under SRA accession numbers SRX14472462, SRX14472461, SRX14472460, SRX14472459, SRX13323315, SRX13323314, SRX13323313, SRX13323312, SRX13323311, SRX13323323, SRX13323320, SRX13323319, SRX13323318, SRX13323310, and SRX13323309 and BioProject accession number PRJNA786454. Genome sequences were deposited in the NCBI GenBank database under accession numbers OP783427 to OP783441 (Table 1).
